# Pediatric WNT medulloblastoma predisposition in intraoperative blood loss: a retrospective observational cohort study

**DOI:** 10.3389/fneur.2024.1386121

**Published:** 2024-07-02

**Authors:** Zaiyu Zhang, Yuxin Wu, Xueling Zhao, Wenyuan Ji, Lusheng Li, Xuan Zhai, Ping Liang, Yuan Cheng, Jianjun Zhou

**Affiliations:** ^1^Department of Neurosurgery, Children's Hospital of Chongqing Medical University, Chongqing, China; ^2^National Clinical Research Center for Child Health and Disorders, Chongqing, China; ^3^Ministry of Education Key Laboratory of Child Development and Disorders, Chongqing, China; ^4^Chongqing Key Laboratory of Child Neurodevelopment and Cognitive Disorders, Chongqing, China; ^5^Department of Neurosurgery, The Second Affiliated Hospital, Chongqing Medical University, Chongqing, China

**Keywords:** blood loss, hemorrhage, medulloblastoma, molecular subgroup, wingless

## Abstract

**Introduction:**

Molecular subgroups influence the vascular architecture within medulloblastomas, particularly the wingless (WNT) subgroup, which contributes to its propensity for primary tumor hemorrhage. Whether this mechanism affects intraoperative blood loss remains unknown. This study aimed to assess the association between WNT medulloblastoma and the predisposition for blood loss.

**Methods:**

This was a retrospective observational study using data from a neuro-oncology center comprising molecular data on patients treated between December 31, 2014, and April 30, 2023. Differences between WNT and other subgroups in the risk of primary outcome-intraoperative blood loss were assessed using multivariable-adjusted linear regression.

**Results:**

Of the 148 patients included in the analysis, 18 patients (12.2%) had WNT, 42 (28.4%) had sonic hedgehog (SHH) *TP53*-wildtype, 7 (4.7%) had SHH *TP53*-mutant, and 81 (54.7%) were non-WNT/ non-SHH. The WNT subgroup more frequently underwent primary intratumoral hemorrhage (22% vs. 3.8%; *p* = 0.011). The median intraoperative blood loss was 400.00 (interquartile range [IQR] 250, 500) mL for WNT and 300.00 [200, 400] mL for the other subgroups (p = 0.136), with an adjusted β of 135.264 (95% confidence intervals [CI], 11.701–258.827; *p* = 0.032). Similar results were observed in both midline and noninfiltrative margin medulloblastoma.

**Discussion:**

WNT medulloblastoma is typically associated with primary intratumoral hemorrhage and intraoperative blood loss. The validity of determining the surgical approach based on predicted molecular subtypes from imaging data is questionable. However, attempting to engage in risk communication with patients in a molecular-specific way is worthwhile to validate.

## 1 Introduction

Medulloblastoma is the most common malignant pediatric brain tumor and the most significant cause of cancer-related morbidity and mortality in this population (1). Currently, the mainstay of management for childhood medulloblastoma remains maximal safe resection followed by risk-adapted craniospinal radiotherapy and chemotherapy (2, 3). Surgical intervention offers the potential benefits of symptom relief, increasing survival, and improving quality of life (4). However, neurosurgical procedures carry the potential for significant intraoperative blood loss, and the resulting allogeneic blood transfusion during neurosurgical procedures exposes patients to immune- or nonimmune-mediated complications (5). There has been increased attention on reducing exposure to allogeneic blood products in children (6, 7).

Understanding the different biology of medulloblastoma subgroups substantially increased over the past years and classified medulloblastoma into at least 4 consensus molecular subgroups: wingless (WNT), sonic hedgehog (SHH) *TP53*-wildtype, SHH *TP53*-mutant, and non-WNT/non-SHH (8). Clinical and radiological characteristics illustrative of a specific subgroup may impact neurosurgical decision-making (9, 10). Recent research has shown that the molecular subgroup influences the intratumoral vessel structure, (11) with WNT medulloblastoma, in particular, being associated with hemorrhagic tumor tissue at diagnosis (9, 12, 13). However, the most recent research that has stratified medulloblastomas into the molecular subgroups has primarily concentrated on survival rates rather than conducting comprehensive analyses of blood loss within the context of these subgroups. Thus, it is probable that patients with these tumors also exhibit varying levels of susceptibility to blood loss, especially the WNT subgroup.

To examine the potential associations mentioned above, we assembled this cohort to interrogate whether there is a predisposition of the WNT subgroup for intraoperative blood loss.

## 2 Materials and methods

### 2.1 Study design and participants

Patients included in this retrospective observational study are a consecutive series of all patients 18 years and younger with a pathological diagnosis of medulloblastoma who were treated with resection from December 31, 2014, until April 30, 2023. We excluded patients with prior surgical histories at external medical facilities. Our study included only those patients with at least a 30-day postoperative follow-up. During the analyzed period, resection procedures were primarily conducted by the 4 specialized onconeurosurgeons, utilizing microsurgical techniques and supported by intraoperative neuromonitoring. Neuronavigation was also employed when necessary. Notably, no significant changes in surgical techniques or technology were used during the period under analysis, eliminating a potential bias within the cohort. This study followed the Strengthening the Reporting of Observational Studies in Epidemiology reporting guidelines (14). Patients or their parents provided written informed consent for tumor collection and examination for biological studies. This study was performed in line with the principles of the Declaration of Helsinki. Approval was granted by the Ethics Committee of our institution (No.2021406).

### 2.2 Molecular studies

Molecular profiling of tumor tissue was performed on a subset of patients who provided informed consent. DNA was extracted from formalin-fixed and paraffin-embedded (FFPE) samples. Low-coverage whole-genome sequencing (LcWGS) was conducted. A panel of 39 genes (*APC*, *ARID1B*, *BCOR*, *CDH1*, *CDK6*, *CHD7*, *CREBBP*, *CSNK2B*, *CTDNEP1*, *CTNNB1*, *DDX31*, *DDX3X*, *GABRG1*, *GFI1*, *GFI1B*, *GLI2*, *GPS2*, *KDM4C*, *KDM6A*, *KMT2B*, *LDB1*, *MLL3*, *MYC*, *MYCN*, *NCOR2*, *OTX2*, *PIK3CA*, *PTCH1*, *PTCH2*, *PTEN*, *SMARCA4*, *SMARCC2*, *SMARCD2*, *SMO*, *SNCAIP*, *SUFU*, *TERT*, *TP53*, *ZMYM3*) was used, and 5 genes (*CDK6*, *GLI2*, *MYC*, *MYCN*, *OTX2*) were screened to analyze copy number variation (CNV). Molecular subgroup classification was predicted based on targeted mutational analysis, CNV, and LcWGS data using unique algorithms such as logistic regression models and random forest models. Genetron Health (Beijing, China) carried out all sequencing and bioinformatics analyses, and the details of these procedures have been previously described (15).

### 2.3 Covariates

Most patient data that were entered prospectively into the Chongqing Higher Institution Engineering Research Center of Children’s Medical Big Data Intelligent Application were retrospectively reviewed. Radiological variables and the use of vasopressors and mannitol in the included patients were retrospectively collected. The preoperative data collected included the following: sex, age, weight, American Society of Anesthesiologists (ASA) score, results of preoperative laboratory examinations (hemoglobin [Hb] level, platelet [PLT] count, prothrombin time [PT], activated partial thromboplastin time [APTT], and fibrinogen [Fib] content), radiological variables (tumor size, tumor location, peritumoral edema, primary intratumoral hemorrhage, tumor margin, and involvement of essential vessels), and history of preoperative cerebrospinal fluid (CSF) diversion. The standard laboratory values of coagulation measured in this study were 9.8–13.3 s for PT, 25.0–35.4 s for APTT, and 1.22–3.89 g/L for Fib.

Preoperative computed tomography or magnetic resonance imaging (MRI) were evaluated for the presence of primary intratumoral hemorrhage. Except for 4 patients who underwent emergency resection due to primary intratumoral hemorrhage without preoperative MRI, all patients were assessed with pre- and postoperative MRI, including gadolinium-injected imaging sequences. Images were independently interpreted retrospectively by two experienced neurosurgeons, with discrepancies being resolved by reference to the radiographic reports reviewed by a neuroradiologist. Tumor size was calculated using the ABC/2 formula, in which parameter A represents the maximum diameter of the tumor measured on the axial image, parameter B represents the maximum diameter at 90° to A, and parameter C represents the number of slices of images multiplied by the slice thickness (16). The horizontal location of medulloblastoma was defined as the location of the epicenter of the tumor in the horizontal axis to the midline of the fourth ventricle. The crucial blood vessels encircling the tumor mainly comprised the vertebrobasilar circulatory system. Contrast enhancement was described as proportion of tumor showing uptake of gadolinium, which was classified into <20%, 20–80%, or > 80%, taking similar approaches (10, 17).

Hydrocephalus was diagnosed when the Evans index, the ratio of the maximum width of the frontal horns to the maximum width of the inner table, was determined to be greater than 0.3 (18). The external ventricular drainage (EVD) placement was decided based on the consulting neurosurgeon’s clinical judgment concerning the patient’s condition of the severity of hydrocephalus-induced intracranial hypertension, time until tumor resection, and the anatomical feasibility of EVD insertion.

The intraoperative data collected included the following: minimal body temperature, hemostatic adjunct administration (epsilon aminocaproic acid [EACA] or hemocoagulase atrox [HCA]), continuous infusion of vasopressors, and use of intravenous mannitol. All medulloblastoma resections were performed under general anesthesia. Routine intraoperative monitoring consisted of electrocardiography, invasive arterial blood pressure, capnography, pulse oximetry, body temperature, urinary output catheter, and central venous pressure. Before the data collection began, each lead neurosurgeon established internal hemostatic therapy guidelines. Although the study was observational and patients were not randomly assigned to treatment arms, precautions were taken to avoid treatment and other selection biases. Instead, patients were analyzed according to their predetermined EACA or HCA group in an intention-to-treat-like manner. Additionally, the principal neurosurgeon and attending anesthesiologist conducted separate, secondary analyses based on the as-treated hemostatic adjunct, which was chosen based on intraoperative blood loss and coagulation competence measured by thromboelastography. EACA was administered (20 mg/kg) over 20 min as a loading dose before the skin incision and was followed by a maintenance infusion of 1 mg/kg/h until the completion of the skin suture. HCA was intravenously injected at a dose of 1 U 15 min before the operation.

Use of a vasopressor and mannitol was guided by the neurosurgical anesthesia task force on intraoperative management. Vasopressor assistance was commonly adjusted utilizing dopamine (3–8 μg/[kg*min]) and epinephrine (0.01–0.5 μg/[kg*min]). An infusion of 20% mannitol (1 g/kg) was completed when (intractable) swelling was seen intraoperatively or when swelling was expected (preventive).

### 2.4 Outcomes

The primary outcome was intraoperative blood loss. The primary surgeon and anesthesiologist independently estimated blood loss in a double-blind way (without preoperative molecular confirmation) during each case by subtracting irrigation fluid from the suction aspirate and gravimetrically assessing the soaked sponges and cotton pledgets after surgery by subtracting the surgical material weight before surgery.

The secondary outcomes included blood product requirements, intravenous fluid therapy, surgical and anesthesia time, and the extent of tumor resection. Participants received our institution’s standard protocol for blood transfusion, which adhered to the perioperative transfusion guidelines established by the National Health Commission of the People’s Republic of China (19). Allogeneic blood product transfusion is typically triggered based on several factors, such as intraoperative hemoglobin levels (between 70 and 90 g/L), the extent of blood loss during surgery, the patient’s hemodynamic status, urinary output, and blood coagulation. The surgical fluids used included crystalloids (0.9% saline and compound sodium chloride) and colloids (6% hydroxyethyl starch and 5% human albumin). We analyzed residual tumor size by MRI approximately 72 h postresection (20). Gross total resection was defined as no visible residual tumor tissue and no reactive enhancement. Near total and subtotal resection was defined as the maximum cross-sectional area of residual ≤1.5 cm^2^ and > 1.5 cm^2^, respectively.

### 2.5 Statistical analysis

The above analyses were performed using SPSS (version 25.0) and R 4.2.3. Each statistical test delineated herein was two-sided, with *p* values <0.05 indicating statistically significant differences. We did not calculate a planned sample size *a priori*, as we used a convenience sample. Participants were categorized into 4 groups according to the molecular subgroup. Categorical variables were denoted as numbers (percentages [%]) and assessed via the χ^2^ test or Fisher’s exact test when the criteria for the *χ*^2^ test were unmet. Continuous variables are presented as the mean ± standard deviation (SD) or median (interquartile range [IQR]) and were compared between the groups by utilizing analysis of variance or equivalent nonparametric tests.

Multivariable-adjusted linear regression models were used to determine the association between molecular subgroups and primary outcomes. Effect sizes with 95% confidence intervals (CIs) were recorded. In multivariable analyses, adjustments were made for sex, age, weight, ASA score, Hb, PLT, PT, APTT, Fib, tumor size, tumor location, peritumoral edema, primary intratumoral hemorrhage, tumor margin, and involvement of vessels (Model 1) and sex, age, weight, ASA score, Hb, PLT, PT, APTT, Fib, preoperative CSF diversion, minimal body temperature, hemostatic adjunct administration, and the use of vasopressors and mannitol (Model 2). For the analyses of intraoperative blood loss and allogeneic blood transfusion, we used univariable linear and logistic regression models when appropriate. For these models, multiple imputations with chained equations were used for missing variables (21).

We stratified the patients into subgroups by tumor location and margin and performed tests of interaction. In several sensitivity analyses, we explored the robustness of the results with several sensitivity analyses *post hoc*, including adjustment for additional factors, and after excluding the cases in our sample with coagulopathy. We additionally performed propensity score matching to assess outcomes between the molecular subgroups, with one-to-four matching to the nearest neighbor conducted based on age, weight, preoperative CSF diversion, and the use of EACA, vasopressors, and mannitol.

## 3 Results

### 3.1 Baseline characteristics

**Figure 1:**
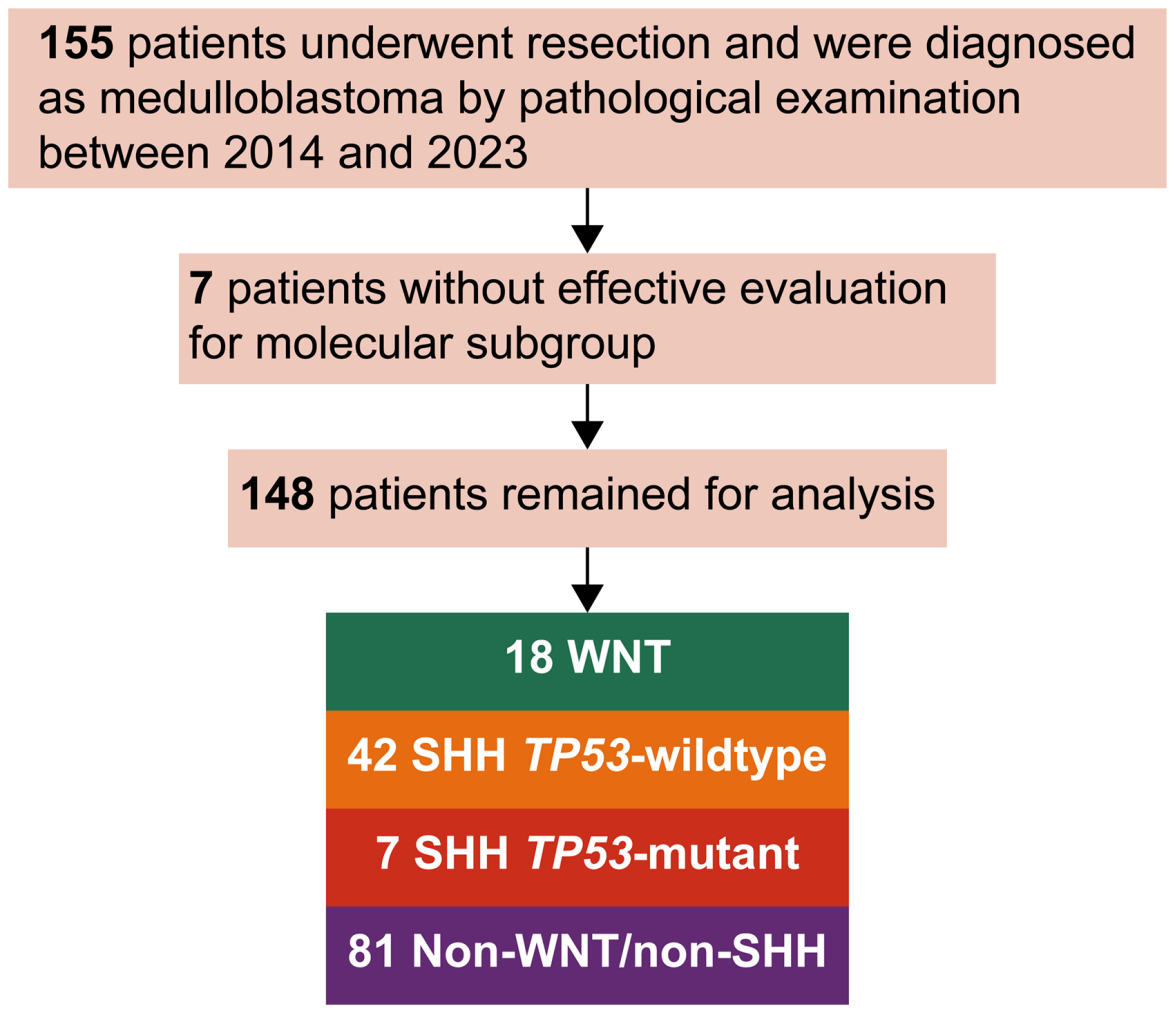
Consort diagram of the study population. SHH, sonic hedgehog; WNT, wingless.

**Figure 2:**
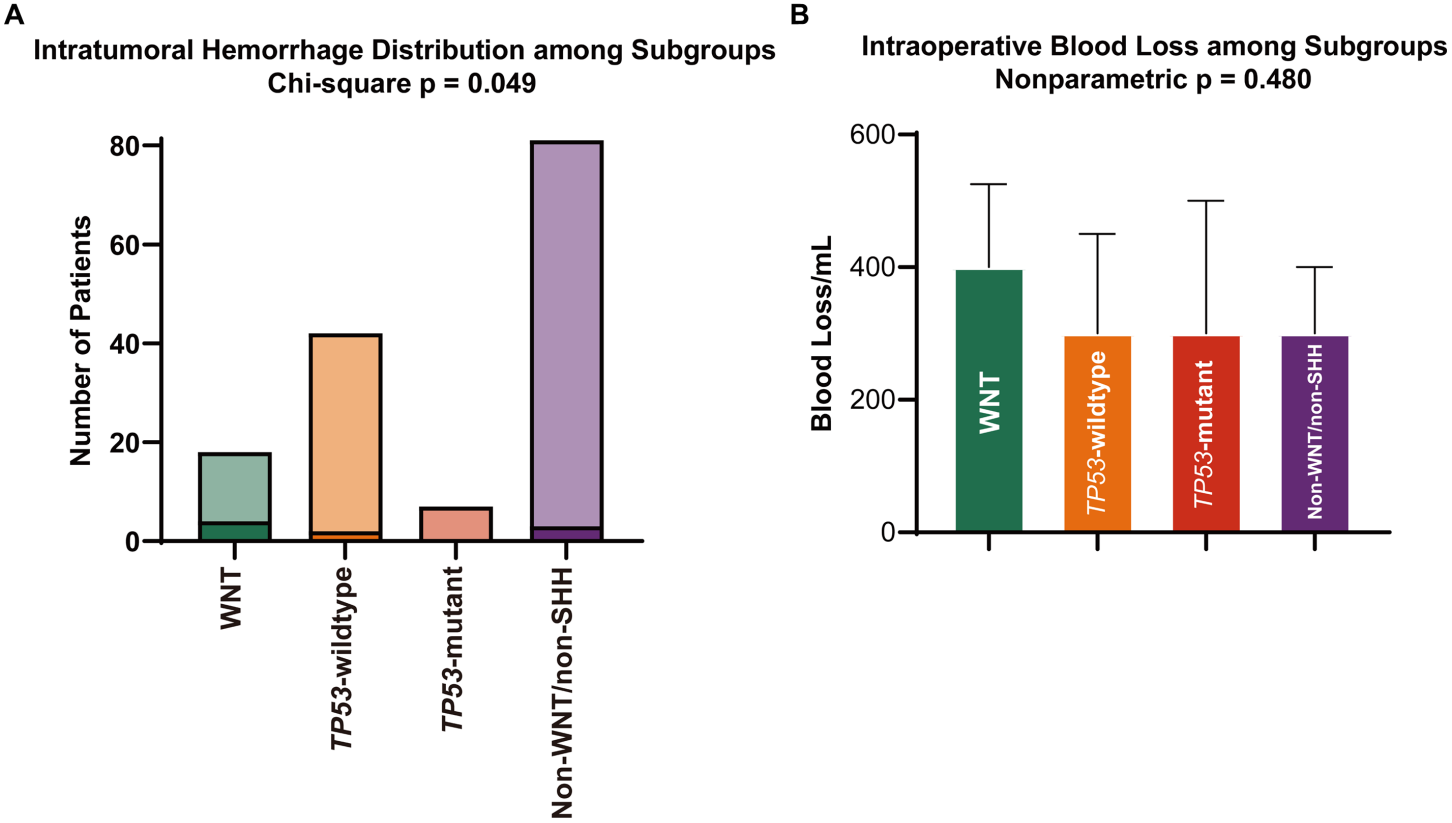
**(A)** Rates of primary intratumoral hemorrhage and **(B)** volume of intraoperative blood loss. SHH, sonic hedgehog; WNT, wingless.

Overall, 148 patients were included in this analysis (Figure 1). Of these, 12.2% were WNT, 28.4% were SHH *TP53*-wildtype, 4.7% were SHH *TP53*-mutant, and 54.7% were non-WNT/non-SHH. The baseline characteristics of each subgroup are summarized in Table 1. Primary intratumoral hemorrhage was observed in 22% (4/18) of patients diagnosed with WNT medulloblastoma but not in significant proportions in other molecular subgroups (3.8% [5/125]; *p* = 0.011; Figure 2A). In addition, there were no fatalities resulting directly from intratumoral hemorrhage.

**Table 1:** Baseline demographic and clinical characteristics by molecular subgroups.

		Molecular subgroup				
Variables	Total = 148	WNT = 18	SHH *TP53*-wildtype = 42	SHH *TP53*-mutant = 7	Non-WNT/non-SHH = 81	*p*-values
Gender, %						0.141
Male	87 (58.8)	6 (33.3)	26 (61.9)	5 (71.4)	50 (61.7)	
Female	61 (41.2)	12 (66.7)	16 (38.1)	2 (28.6)	31 (38.3)	
Age, years	7.09 ± 3.39	9.25 ± 2.42	6.11 ± 3.83	7.77 ± 2.87	7.06 ± 3.19	0.010
Weight, kg	21.00 [16.50, 28.00]	25.25 [21.12, 29.00]	20.00 [15.12, 23.00]	22.00 [17.50, 29.00]	21.00 [17.50, 27.00]	0.046
ASA score, %						0.107
II	9 (6.1)	0 (0.0)	0 (0.0)	0 (0.0)	9 (11.1)	
III	124 (83.8)	17 (94.4)	35 (83.3)	6 (85.7)	66 (81.5)	
IV	15 (10.1)	1 (5.6)	7 (16.7)	1 (14.3)	6 (7.4)	
Hb, g/L	127.27 ± 12.74	127.11 ± 15.12	125.57 ± 13.93	133.43 ± 11.12	127.65 ± 11.67	0.487
PLT, 10^9^/L	304.74 ± 87.24	283.67 ± 88.09	329.07 ± 94.78	304.14 ± 127.44	296.85 ± 77.73	0.173
PT, second	11.30 [10.80, 11.80]	11.40 [10.80, 11.67]	11.20 [10.70, 11.67]	10.80 [10.15, 11.30]	11.30 [10.90, 11.90]	0.100
APTT, second	28.30 [26.10, 30.33]	28.00 [26.50, 29.20]	27.35 [25.73, 29.78]	29.00 [22.40, 30.95]	28.60 [26.50, 30.40]	0.616
Fib, g/L	2.17 [1.90, 2.57]	2.16 [1.92, 2.66]	2.31 [1.89, 2.74]	2.08 [1.77, 2.49]	2.16 [1.91, 2.46]	0.602
Tumor size, cm^3^	31.14 [21.76, 43.69]	30.93 [19.75, 36.84]	33.91 [23.03, 57.08]	16.73 [16.29, 23.13]	31.38 [23.68, 40.45]	0.105
Midline location, %	114 (77.0)	17 (94.4)	25 (59.5)	2 (28.6)	70 (86.4)	< 0.001
Peritumoral edema, %	105 (70.9)	14 (77.8)	25 (59.5)	5 (71.4)	61 (75.3)	0.291
Primary intratumoral hemorrhage, %	9 (6.1)	4 (22.2)	2 (4.8)	0 (0.0)	3 (3.7)	0.049
Infiltrative margin, %	24 (16.2)	1 (5.6)	12 (28.6)	3 (42.9)	8 (9.9)	0.007
Involvement of vessels, %	10 (6.8)	1 (5.6)	6 (14.3)	0 (0.0)	3 (3.8)	0.179
Contrast enhancement, %						0.002
<20%	35 (23.6)	1 (5.6)	3 (7.1)	1 (14.3)	30 (37.0)	
20–80%	50 (33.8)	6 (33.3)	17 (40.5)	3 (42.9)	24 (29.6)	
>80%	63 (42.6)	11 (61.1)	22 (52.4)	3 (42.9)	27 (33.3)	
Preoperative CSF diversion, %	114 (77.0)	14 (77.8)	27 (64.3)	4 (57.1)	69 (85.2)	0.028
Minimal body temperature, °C	35.30 [35.00, 35.90]	35.45 [35.00, 35.77]	35.20 [35.00, 35.82]	35.35 [35.20, 35.80]	35.30 [35.00, 36.00]	0.914
Hemostatic adjunct administration, %						
EACA	21 (14.2)	6 (33.3)	7 (16.7)	2 (28.6)	6 (7.4)	0.013
HCA	111 (75.0)	10 (55.6)	35 (83.3)	5 (71.4)	61 (75.3)	0.143
Continuous infusion of vasopressors, %	114 (77.0)	18 (100.0)	29 (69.0)	6 (85.7)	61 (75.3)	0.031
Use of mannitol, %	14 (9.5)	1 (5.6)	9 (21.4)	1 (14.3)	3 (3.7)	0.011

Data are expressed as the mean ± SD, median [25th, 75th percentile] or number (%). All percentages reflect denominators encompassing known or reported values, which do not always equate to the total number of the cohort. Percentages may not add up to 100% because of rounding. The *p* values indicate the *χ*^2^ test for categorical variables, the analysis of variance for age, PLT, and Hb, and the nonparametric test for weight, PT, APTT, Fib, tumor size, and minimal body temperature. APTT, activated partial thromboplastin time; ASA, American Society of Anesthesiologists; CSF, cerebrospinal fluid; EACA, epsilon aminocaproic acid; Fib, fibrinogen content; Hb, hemoglobin; HCA, hemocoagulase atrox; PLT, platelet count; PT, prothrombin time; SD, standard deviation; SHH, sonic hedgehog; WNT, wingless.

Given our research aims and the distinct vascular structure, as well as preoperative hemorrhage tendencies in WNT medulloblastoma, we sought to divide medulloblastoma into 2 groups: WNT and other subgroups. Compared with patients in other subgroups, patients in WNT were more often female (12 of 18 [66.7%] vs. 49 of 130 [37.7%]; *p* = 0.037), were older (mean ± SD, 9.25 ± 2.42 years vs. 6.79 ± 3.41 years; *p* < 0.004), and had often a higher weight (median [IQR], 25.25 [21, 29] kg vs. 21.00 [16, 26] kg; *p* = 0.020). The vast majority (94.4%) of patients in the WNT subgroup exhibited significant contrast enhancement, with 61.1% of patients showing uptake in >80% of the tumor volume, although this proportion did not exhibit statistically significant differences when compared to other molecular subgroups of medulloblastoma (*p* = 0.106). Patients with WNT medulloblastoma more frequently underwent EACA (6 of 18 [33.3%] vs. 15 of 130 [11.5%]; *p* = 0.034) and had no continuous infusion of vasopressors (0 of 18 vs. 34 of 130 [26.2%]; *p* = 0.030).

### 3.2 WNT and intraoperative blood loss

The blood loss volume was a more significant trend in the WNT subgroup, but it was not statistically significant (median [IQR], 400.00 [250, 500] mL vs. 300.00 [200, 400] mL; *p* = 0.136; Figure 2B, Table 2). There were no differences in the transfusion of allogeneic blood products, the infusion volume of crystalloids and colloids, the surgical and anesthesia time, or the resection extent (Table 2). For all subgroups, intraoperative blood loss was associated with an increased transfusion volume of red blood cells (β, 0.054; 95% CI, 0.044–0.064; *p* < 0.001), transfusion of fresh frozen plasma (odds ratio [OR], 1.007; 95% CI, 1.004–1.010; *p* < 0.001) and cryoprecipitation (OR, 1.006; 95% CI, 1.002–1.010; *p* = 0.003).

**Table 2:** Primary and secondary outcomes by molecular subgroups.

		Molecular subgroup (Number)				
Outcomes	Total (148)	WNT (18)	SHH *TP53*-wildtype (42)	SHH *TP53*-mutant (7)	Non-WNT/non-SHH (81)	*p*-values
Blood loss, mL	300.00 [200.00, 462.50]	400.00 [250.00, 500.00]	300.00 [200.00, 437.50]	300.00 [225.00, 400.00]	300.00 [200.00, 400.00]	0.480
RBC transfusion, ml/kg	10.17 [0.00, 20.26]	9.35 [0.00, 17.08]	12.60 [8.33, 26.52]	13.33 [4.55, 20.71]	9.52 [0.00, 17.39]	0.151
Fresh frozen plasma transfusion, %	29 (19.6)	3 (16.7)	9 (21.4)	1 (14.3)	16 (19.8)	0.983
Cryoprecipitate transfusion, %	5 (3.4)	1 (5.6)	2 (4.8)	0 (0.0)	2 (2.5)	0.624
Crystalloids infusion, ml/kg	70.42 [50.00, 92.11]	69.88 [52.21, 93.80]	73.51 [52.26, 92.22]	73.21 [59.44, 84.17]	67.27 [45.65, 90.00]	0.647
Colloid infusion, ml/kg	11.54 [8.70, 16.85]	11.88 [9.71, 16.70]	12.50 [9.09, 17.24]	12.50 [10.13, 18.33]	11.11 [8.70, 15.79]	0.905
Surgical time, minutes	392.50 [320.75, 465.00]	375.50 [338.25, 459.25]	393.00 [295.50, 473.00]	410.00 [350.00, 428.00]	395.00 [330.00, 475.00]	0.939
Anesthesia time, minutes	490.00 [416.00, 571.25]	493.00 [437.00, 575.00]	490.00 [385.25, 570.00]	510.00 [450.00, 527.50]	490.00 [420.00, 591.00]	0.925
Extent of resection, %						0.136
Gross total resection	120 (81.1)	15 (83.3)	38 (90.5)	4 (57.1)	63 (77.8)	
Near total resection	10 (6.8)	2 (11.1)	1 (2.4)	0 (0.0)	7 (8.6)	
Subtotal resection	18 (12.2)	1 (5.6)	3 (7.1)	3 (42.9)	11 (13.6)	

Data are expressed as the median [25th, 75th percentile] or number (%). All percentages reflect denominators encompassing known or reported values, which do not always equate to the total number of the cohort. Percentages may not add up to 100% because of rounding. The *p* values indicate the χ^2^ test for categorical variables and the nonparametric test for continuous variables of nonnormal distribution. RBC, red blood cell; SHH, sonic hedgehog; WNT, wingless.

In the linear regression models (Table 3), WNT medulloblastoma was associated with significantly more intraoperative blood loss in both the unadjusted (β, 163.721; 95% CI, 40.526–286.916; *p* = 0.009) and adjusted analyses (adjusted β [aβ], 125.088; 95% CI, 8.714–241.463; *p* = 0.035 for Model 1 and aβ, 135.264; 95% CI, 11.701–258.827; *p* = 0.032 for Model 2). The association was similar in the midline medulloblastoma (unadjusted β, 176.537; 95% CI, 43.353–309.721; *p* = 0.009; aβ, 135.712; 95% CI, 7.838–263.585; *p* = 0.038 for Model 1 and aβ, 158.771; 95% CI, 26.478–291.065; *p* = 0.019) and noninfiltrative margin groups (unadjusted β, 164.150; 95% CI, 29.658–298.641; *p* = 0.017 and aβ, 157.453; 95% CI, 21.932–292.974; *p* = 0.023 for Model 2; Table 3).

**Table 3:** Linear regression for blood loss.

	*β* (95% CI)	*p*-values	No. (WNT, other subgroups)
Total patients			18, 130
Unadjusted	163.721 (40.526, 286.916)	0.009	
Adjusted Model 1	125.088 (8.714, 241.463)	0.035	
Adjusted Model 2	135.264 (11.701, 258.827)	0.032	
Midline medulloblastoma			17, 97
Unadjusted	176.537 (43.353, 309.721)	0.009	
Adjusted Model 1	135.712 (7.838, 263.585)	0.038	
Adjusted Model 2	158.771 (26.478, 291.065)	0.019	
Lateral medulloblastoma			1, 33
Unadjusted	−200.641 (−1185.593, 784.311)	0.690	
Adjusted Model 1	−546.137 (−1424.937, 332.662)	0.223	
Adjusted Model 2	119.919 (−604.053, 843.890)	0.745	
Location interaction p	0.384		
Infiltrative margin			1, 23
Unadjusted	452.389 (−294.215, 1198.993)	0.235	
Adjusted Model 1	431.629 (−287.394, 1150.651)	0.239	
Adjusted Model 2	74.147 (−381.908, 530.202)	0.750	
Noninfiltrative margin			17, 107
Unadjusted	164.150 (29.658, 298.641)	0.017	
Adjusted Model 1	124.376 (−2.306, 251.059)	0.054	
Adjusted Model 2	157.453 (21.932, 292.974)	0.023	
Margin interaction *p*	0.871		
Propensity score matching			
Unadjusted	196.486 (71.245, 321.728)	0.002	14, 45
Adjusted Model 2 with additional factors			18, 130
Model 2 with tumor size	139.942 (21.881, 258.003)	0.020	
Model 2 with peritumoral edema	135.195 (11.388, 259.002)	0.032	
Model 2 with intratumoral hemorrhage	151.506 (22.268, 280.743)	0.022	
Model 2 with the involvement of vessels	133.789 (12.373, 255.206)	0.031	
Model 2 with coagulopathy excluded	139.990 (16.819, 263.161)	0.026	18, 125

The wingless (WNT) subgroup is the reference category. Model 1 was adjusted for sex, age, weight, American Society of Anesthesiologists score, hemoglobin, platelet count, prothrombin time, activated partial thromboplastin time, fibrinogen content, tumor size, tumor location, peritumoral edema, intratumoral hemorrhage, tumor margin, and vessel involvement. Model 2 was adjusted for sex, age, weight, American Society of Anesthesiologists score, hemoglobin, platelet count, prothrombin time, activated partial thromboplastin time, fibrinogen content, preoperative cerebrospinal fluid (CSF) diversion, minimal body temperature, hemostatic adjunct administration, and the use of vasopressors and mannitol. One-to-four matching to the nearest neighbor was conducted based on age, weight, preoperative CSF diversion, and the use of epsilon aminocaproic acid, vasopressors, and mannitol.

In the propensity score–matched cohort, the patient characteristics were similar concerning those factors following one-to-four matching. The association between increased β of intraoperative blood loss and WNT medulloblastoma remained (Table 3). The results of increased intraoperative blood loss in WNT subgroup surgeries were consistent when tumor size, peritumoral edema, primary intratumoral hemorrhage, or involvement of vessels were added to Model 2 or when patients with coagulopathy were removed from the sample (Table 3).

## 4 Discussion

Our single-center, retrospective, observational cohort found that WNT in routine neurosurgical practice correlates with an escalation in intraoperative blood loss compared to other subgroups. The adjusted effect size of blood loss was approximately 150 mL higher than that for the other molecular subgroups. Tumor location and margin did not significantly modify the association between the WNT subgroup and blood loss (interaction *p* = 0.384 and 0.871, respectively; Table 3), but this effect size increase was not consistent in lateral and infiltrative margin medulloblastoma. The lower proportions and the relatively small sample in the WNT subgroup likely explain the nominal β values in the lateral and infiltrative margin groups. The WNT groups had more significant recognized risk factors for blood loss at baseline (i.e., age, weight, preoperative CSF diversion, administration of EACA, and infusion of vasopressor) (22–25). This bleeding risk was consistent in the propensity score–matched cohort, which was utilized to create 2 matched cohorts to minimize these potential confounding factors. We repeated the analyses after adjusting for variables and excluding participants to minimize the effects of the biological features of medulloblastoma. However, the association between WNT and increased blood loss did not change.

Recently, 2 independent cohorts of 99 and 111 medulloblastoma patients were evaluated, in which no differences could be determined among the molecular subgroups with respect to primary intratumoral hemorrhage (10, 17). In contrast, the study from Reisinger et al. also found a numerically higher incidence of primary intratumoral hemorrhage in WNT that was consistent with our observation, and that study showed that there was a relatively infrequent incidence in non-WNT and that there were no patients in a review of non-WNT patients (9). Additionally, in several reported cases of the WNT subgroup, a higher incidence of hemorrhage was observed at the time of diagnosis compared to other subgroups (12, 13). Despite the rarity of primary intratumoral hemorrhage in medulloblastoma, and within our cohort, no patients died directly due to primary intratumoral hemorrhage during acute intervention and subsequent tumor resection; it is crucial to recognize that it represents a serious and potentially fatal condition in a substantial number of cases (26–28). Overall, we agreed to evaluate radiological indicators of primary intratumoral hemorrhage as biomarkers for WNT medulloblastoma. However, such explorations for this relationship can be made in the future with standardized protocols and controlled designs, possibly with randomization, which may affect discrimination and concordance of the radiologic prediction.

Within our study cohort, the intraoperative blood loss in SHH and non-WNT/non-SHH medulloblastoma showed a discernible disparity compared to the WNT subgroup. In radiological analyses, we did not observe differences in the number of vascular supplies of WNT-medulloblastomas in terms of contrast enhancement and relationship with important blood vessels. However, there is evidence suggesting that the vascular architecture supplying WNT-medulloblastomas may exhibit certain distinctive features. Although the mechanism behind the association of WNT medulloblastoma with blood loss remains unclear, there is evidence that WNT medulloblastoma possessing an aberrant, non-CNS vasculature has a direct positive effect on hemorrhage that exhibits a distinct pattern and differs from the other subgroups (11). Preclinical studies have indicated that paracrine signals caused by mutant β-catenin in WNT medulloblastoma cells can affect vascular development by altering gene expression in endothelial cells, resulting in blood–brain barrier dysfunction and leading to increased intratumoral hemorrhage. Additionally, the involvement of the WNT signaling pathway in vascularization has further implications, which may also impact hemorrhagic susceptibility (29, 30). However, our study did not demonstrate differences in vascular density among WNT subgroups at the pathological level, highlighting the need for further research in this field.

Significant amounts of blood loss may occur during craniotomy procedures, which can cause these patients to frequently require allogeneic blood transfusion, (31) consistent with our results. However, the results showed that although the blood loss volume was estimated to be higher in the WNT subgroup, it did not lead to differences in things that matter, such as transfusion of blood products, intravenous fluid therapy, operating and anesthesia time and extent of resection. This reminds us that if perioperative teams approach these cases with the standard caution one would expect, the impact of pathological subgroups on intraoperative or postoperative outcomes can be minimized. Therefore, the neurosurgeon and anesthesiologists should be well equipped to anticipate and manage intraoperative blood loss for all patients, regardless of the suspected molecular subgroup.

Our data indicate that intraoperative blood loss is primarily influenced by an endogenous factor of medulloblastoma, the molecular subgroup, which seems to present a contradiction in terms of temporal logic. However, these blood loss risks reflect the typical spectrum of characteristics seen in patients and tumors. However, a well-balanced discussion about surgical risks should be done with all patients/families regardless of the suspected pathology. The provision of information has the potential to enhance the extensive knowledge of the neuro-oncology team, and this enhanced knowledge can be utilized during discussions about surgical procedures with patients and their parents. The potential of personalized risk communication in a subgroup-specific manner for neurosurgeries is worthy of further verification in future investigations.

Currently, the prognostic importance of the extent of resection for WNT medulloblastoma is unclear and warrants re-evaluation, (4) especially considering the well-established favorable prognosis of the WNT subgroup (32, 33). Based on previous documentaries, (23, 34) it is crucial to consider the detrimental effects of intraoperative blood loss on the neurosurgical outcome. Thus, a function-sparing and bleeding-minimal near total excision could be preferred to achieve the best possible outcome. Additionally, WNT-tumors originate from the brainstem, often located within the fourth ventricle. Their arterial blood supply mainly stems from the vermian branches of the posterior inferior cerebellar artery, whereas SHH- tumors are typically situated in the cerebellar hemispheres, with their arterial supply primarily from hemispheric branches. The feeding arteries of WNT-medulloblastomas are closer to the midline, potentially placing them in closer proximity to the vertebral arteries, brainstem, and cranial nerves. Based on this, our tentative recommendation is that if preoperative imaging suggests a possible WNT medulloblastoma and during the resection process, achieving gross total resection poses challenges due to critical neural and vascular structures, subtotal resection is also deemed acceptable. From this certain perspective, it seems reasonable to leave behind residual tumor tissue if the tumor exhibits a propensity for hemorrhage and the surgeon encounters significant bleeding during the operation. However, these residual tumor tissues with a tendency to bleed undoubtedly increase the risk of postoperative hemorrhage, which is certainly more critical than intraoperative bleeding (35, 36).

Although advances in molecular profiling have led to changes in adjuvant medical treatment for childhood medulloblastoma, maximal safe surgical resection remains a cornerstone of neurosurgical management. However, both Reisinger et al. (9) and Phoenix et al. (11) suggested that distinguishing WNT medulloblastoma by the neuroimaging presence of significant hemorrhage in the fourth ventricle or arising exclusively from the brainstem is advantageous, as a less aggressive and potentially debilitating resection, followed by radio- and chemotherapy, could lead to excellent survival rates in this subset. Nevertheless, in view of the current limited ability of imaging prediction, (10, 17) and when faced with this decision, especially considering that primary intratumoral hemorrhage and characteristic spatial predilection should raise hints of a WNT medulloblastoma and lead to a less aggressive surgical strategy, it is likely that every neurosurgeon may ask themselves, “How sure can a surgeon reasonably be?” The odds might be higher, but would that truly change the goals of surgery? Substantially, it is still too early for neurosurgeons to gradually shift toward more conservative resection strategies in cases where imaging features suggest a WNT medulloblastoma. The application of artificial intelligence and radiomics, such as using large contrast agents to test whether WNT-medulloblastomas are associated with a unique, blood brain barrier-penetrant image phenotype, (11) is expected to move the field forward (37, 38).

There are significant limitations to our analysis. First, the retrospective and nonrandomized nature of the analysis introduces potential bias; we did not have a central blinded assessment of the intraoperative blood loss. Second, as these results are limited to the surgical experience of our team, they may not necessarily represent broader patterns across all neurosurgery centers. Third, we could not determine the impact of the WNT subgroup on blood loss in relatively small sample groups, such as patients with lateral or infiltrative margin tumors. Finally, the association between WNT and blood loss was not significant in the equivalent nonparametric test but became significant in the multivariable linear regression. We hypothesize that the main reason is confounding. Although we have tried to minimize the impact of confounding using various statistical methods, such as adjusting variables according to the clinical analysis, performing interaction analyses, and propensity score matching, there may still be some potential confounders or their combined effects that have influenced the results. Nevertheless, overall, the results of the final multivariable analysis have been shown to be quite reliable. We look forward to validating these findings in future related prospective, large-sample studies.

Our findings confirmed another significant clinical disparity within the 4 core medulloblastoma subgroups. WNT medulloblastoma is typically associated with significant primary intratumoral hemorrhage at the time of diagnosis and intraoperative blood loss. It is debatable that intratumoral hemorrhage and radiological characteristic spatial predilection in a posterior fossa tumor could raise suspicion of the WNT subgroup and guide the neuro-oncology team toward a less aggressive resection. However, with well-equipped anticipation and well-balanced discussion, doctor–patient/family communication about surgical risks in a molecular-specific manner is indeed worth further verification in the future.

## Data availability statement

The data presented in the study are deposited in the NCBI Sequence Read Archive repository, accession number PRJNA1124362.

## Ethics statement

The studies involving humans were approved by the Ethics Committee of the Children’s Hospital of Chongqing Medical University. The studies were conducted in accordance with the local legislation and institutional requirements. Written informed consent for participation in this study was provided by the participants’ legal guardians/next of kin.

## Author contributions

ZZ: Writing – review & editing, Writing – original draft, Visualization, Software, Methodology, Investigation, Formal analysis, Data curation, Conceptualization. YW: Writing – review & editing, Writing – original draft, Visualization, Software, Methodology, Investigation, Formal analysis, Data curation, Conceptualization. XueZ: Writing – review & editing, Validation, Resources, Investigation, Formal analysis, Conceptualization. WJ: Writing – review & editing, Validation, Resources, Investigation, Formal analysis, Conceptualization. LL: Writing – review & editing, Validation, Supervision, Resources, Project administration, Investigation, Formal analysis, Conceptualization. XuaZ: Writing – review & editing, Validation, Supervision, Resources, Project administration, Investigation, Formal analysis, Conceptualization. PL: Writing – review & editing, Validation, Supervision, Resources, Project administration, Investigation, Formal analysis, Conceptualization. YC: Writing – review & editing, Validation, Supervision, Resources, Project administration, Investigation, Formal analysis, Conceptualization. JZ: Writing – review & editing, Validation, Supervision, Resources, Project administration, Investigation, Formal analysis, Conceptualization.
